# Palladium-catalyzed *ortho*-halogenations of acetanilides with *N*-halosuccinimides via direct sp^2^ C–H bond activation in ball mills

**DOI:** 10.3762/bjoc.14.31

**Published:** 2018-02-16

**Authors:** Zi Liu, Hui Xu, Guan-Wu Wang

**Affiliations:** 1 Hefei National Laboratory for Physical Sciences at Microscale and Department of Chemistry, University of Science and Technology of China, Hefei, Anhui 230026, P. R. China; 2 State Key Laboratory of Applied Organic Chemistry, Lanzhou University, Lanzhou, Gansu 730000, P. R. China

**Keywords:** acetanilide, ball milling, C–H activation, halogenation, mechanochemistry, *N*-halosuccinimide, palladium catalysis

## Abstract

A solvent-free palladium-catalyzed *ortho*-iodination of acetanilides using *N*-iodosuccinimide as the iodine source has been developed under ball-milling conditions. This present method avoids the use of hazardous organic solvents, high reaction temperature, and long reaction time and provides a highly efficient methodology to realize the regioselective functionalization of acetanilides in yields up to 94% in a ball mill. Furthermore, the current methodology can be extended to the synthesis of *ortho*-brominated and *ortho*-chlorinated products in good yields by using the corresponding *N*-halosuccinimides.

## Introduction

Aryl halides have been widely utilized in organic syntheses, which give access to a range of complex natural products [1–2]. However, traditional halogenations of aromatic compounds by direct electrophilic halogenation [3] and Sandmeyer reaction [4] have several drawbacks such as low regioselectivities, complicated reaction procedures and even a risk of danger. Thus, it is necessary to discover new approaches to the regioselective construction of C–X bonds. With the development of transition-metal-catalyzed cross-coupling reactions, a series of halogenations at the *ortho*-position of the directing groups have been disclosed [5–18]. Nevertheless, from the viewpoint of green chemistry, the reduction or even elimination of organic solvents, shorter reaction times, simplification of work-up procedures and improvement of product yields are highly demanding. In recent years, the application of mechanochemical techniques in organic syntheses has attracted increasing attention [19–28].

A few mechanochemical *ortho*-C–H bond activation reactions under the catalysis of rhodium and palladium salts have been reported [29–38]. Hernández and Bolm reported the rhodium-catalyzed bromination and iodination of 2-phenylpyridine using *N*-bromosuccinimide (NBS) and *N*-iodosuccinimide (NIS), respectively, as the halogen source [30]. However, the mechanochemical *ortho*-halogenation using the cheaper palladium catalysts has not been reported yet. In continuing our interest in mechanochemistry [21–22,39–41] and C–H activation reactions [42–44], we have independently investigated the solvent-free *ortho*-iodination of acetanilides under ball-milling conditions [45]. In addition, the current reaction can be extended to *ortho*-bromination and *ortho*-chlorination by using the corresponding *N*-halosuccinimides. Herein, we report these regioselective *ortho*-halogenations in detail.

## Results and Discussion

To begin our study, *N*-(*p*-tolyl)acetamide (**1a**) was chosen as the model substrate to react with NIS using Pd(OAc)_2_ as the catalyst to optimize reaction parameters such as additive, reaction time and reagent ratio. The reaction of **1a** (0.4 mmol) with NIS (0.4 mmol) was initially performed under the catalysis of Pd(OAc)_2_ (10 mol %) in a Spex SamplePrep 8000 mixer mill at a frequency of 875 cycles per minute at room temperature for 3 h. Unfortunately, the desired iodinated product was not detected (Table 1, entry 1). Then, various acids were examined because the addition of acids into the reaction system could promote the C–H bond halogenation according to the previous literature [46]. As desired, compound **2a** was isolated in 87% yield when *p*-toluenesulfonic acid (PTSA) was employed (Table 1, entry 2). A control experiment was conducted for the reaction of **1a** with NIS in the absence of Pd(OAc)_2_, yet still with PTSA as the promoter, and no iodinated product was furnished (Table 1, entry 3). The use of D-camphorsulfonic acid (D-CSA) or mesitylenesulfonic acid dihydrate provided inferior results than that obtained in the presence of PTSA (Table 1, entries 4 and 5 vs entry 2). Furthermore, no desired product was obtained when pyridine-2-sulfonic acid, 2-nitrobenzoic acid, 2-aminoethanesulfonic acid or tungstophosphoric acid hydrate (HPA) was used in the reaction (Table 1, entries 6–9). Thus, the combination of Pd(OAc)_2_ with PTSA was essential for the reaction to take place effectively. Subsequently, the ratio of substrates was investigated, and the results demonstrated that the amount of both NIS and PTSA affected the product yield. Decreasing or increasing the amount of PTSA was not beneficial to the reaction (Table 1, entries 10 and 11). When the amount of NIS was increased from 1.0 equiv to 1.5 equiv and 2.0 equiv, the yield of the iodinated product did not further go up (Table 1, entries 12 and 13). The iodination was slightly less efficient for a shorter time of 2 h (Table 1, entry 14), and prolongation of the reaction time from 3 h to 4 h did not lead to a superior result (Table 1, entry 15).

**Table 1:** Optimization of the reaction conditions.^a^

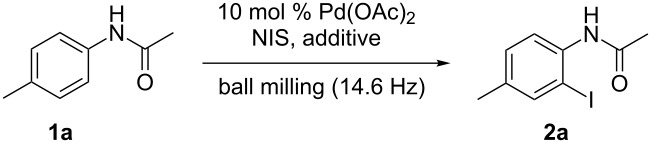				

entry	ratio of reagents^b^	additive	time (h)	yield^c^ (%)

1	1:0.1:1:0	–	3 h	N.R.
**2**	**1:0.1:1:2**	**PTSA**	**3 h**	**87**
3	1:0:1:2	PTSA	3 h	N.R.
4	1:0.1:1:2	D-CSA	3 h	62
5	1:0.1:1:2	mesitylenesulfonic acid dihydrate	3 h	56
6	1:0.1:1:2	pyridine-2-sulfonic acid	3 h	N.R.
7	1:0.1:1:2	2-nitrobenzoic acid	3 h	N.R.
8	1:0.1:1:2	2-aminoethanesulfonic acid	3 h	N.R.
9	1:0.1:1:2	HPA	3 h	N.R.
10	1:0.1:1:1.5	PTSA	3 h	81
11	1:0.1:1:2.5	PTSA	3 h	86
12	1:0.1:1.5:2	PTSA	3 h	88
13	1:0.1:2:2	PTSA	3 h	86
14	1:0.1:1:2	PTSA	2 h	80
15	1:0.1:1:2	PTSA	4 h	87

^a^Unless otherwise specified, all the reactions were carried out in a Spex SamplePrep 8000 mixer mill using **1a** (0.4 mmol). ^b^The reagent ratio referred to **1a**:Pd(OAc)_2_:NIS:additive. ^c^Isolated yield. N.R. = no reaction.

To demonstrate the generality of this protocol, the regioselective iodination of a series of acetanilides was then examined in the presence of Pd(OAc)_2_ and PTSA under the ball-milling conditions (Table 2). Gratifyingly, the *ortho*-iodinated acetanilides were obtained in moderate to good isolated yields. Both *p*-Me and *m*-Me-substituted acetanilides provided products **2a** and **2b** in excellent yields of 87% and 80%, respectively (Table 2, entries 1 and 2). As expected, 3,4-dimethylacetanilide underwent iodination successfully at the less sterically hindered *ortho*-position and gave product **2c** in 85% yield (Table 2, entry 3). The unsubstituted acetanilide provided the desired product **2d** in 77% yield (Table 2, entry 4). It is worth mentioning that the presence of a potentially reactive group, such as fluoro, chloro, and bromo substituents in the acetanilides was tolerable, and products **2e**–**i** were isolated in 51–94% yields (Table 2, entries 5–9), highlighting the functional group compatibility of the current protocol. The presence of an acetyl group at the *para*-position of the phenyl ring of acetanilide **1j** decreased the yield of the corresponding product **2j** to 11% (Table 2, entry 10). Unfortunately, substrates bearing a strong electron-donating methoxy group and a strong electron-withdrawing nitro group could not afford any desired products, and the reason is not quite clear right now.

**Table 2:** Substrate scope.^a^

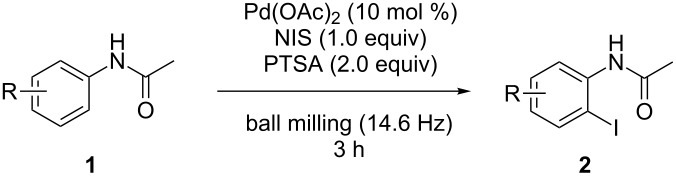			

entry	substrate **1**	product **2**^b^	yield^c^ (%)

1	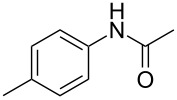 **1a**	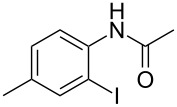 **2a**	87
2	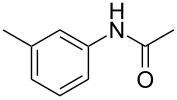 **1b**	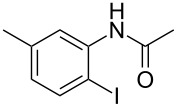 **2b**	80
3	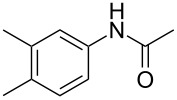 **1c**	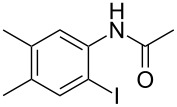 **2c**	85
4	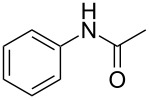 **1d**	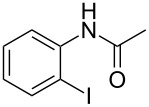 **2d**	77
5	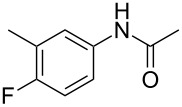 **1e**	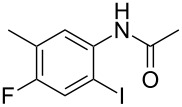 **2e**	94
6	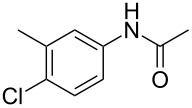 **1f**	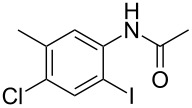 **2f**	71
7	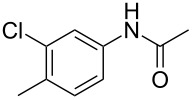 **1g**	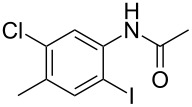 **2g**	74
8	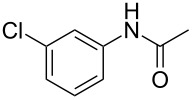 **1h**	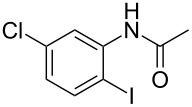 **2h**	51
9	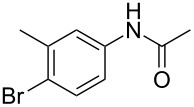 **1i**	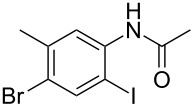 **2i**	70
10	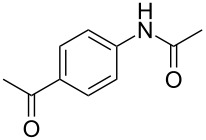 **1j**	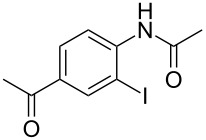 **2j**	11

^a^All the reactions were carried out in a Spex SamplePrep 8000 mixer mill using **1** (0.4 mmol), NIS (0.4 mmol), Pd(OAc)_2_ (10 mol %) and PTSA (0.8 mmol) for 3 h. ^b^Properly characterized by ^1^H NMR, ^13^C NMR, and HRMS spectral data. ^c^Isolated yield.

**Scheme 1:**
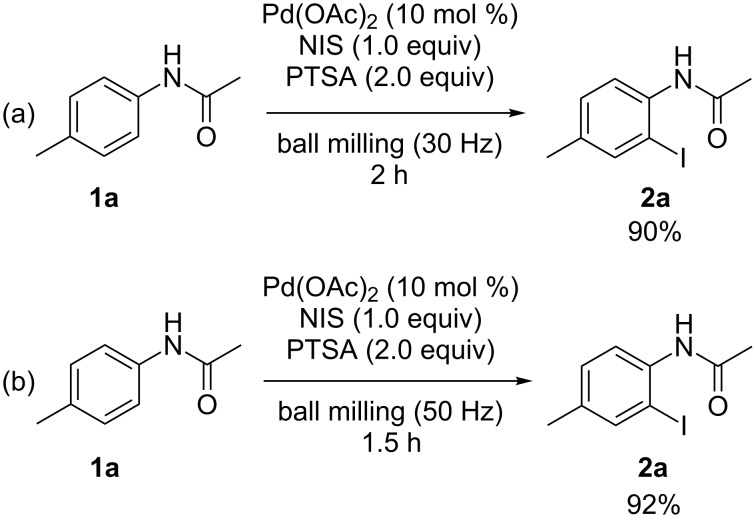
The influence of the milling frequency on the reaction of **1a** with NIS.

In an aim to investigate the influence of the milling frequency, the model reaction of **1a** with NIS was conducted by employing different types of mixer mills with different milling frequencies. *Ortho*-iodized acetanilide **2a** was furnished in 90% yield after milling for 2 h by using a Retsch MM 200 mixer mill (30 Hz, Scheme 1a). At a milling frequency of 50 Hz in a Spex SamplePrep 5100 mixer mill, the iodination was accomplished within 1.5 h to afford the corresponding product **2a** in 92% yield (Scheme 1b). According to the above experimental results, it could be concluded that the higher milling frequency had a beneficial effect on the reaction efficiency in terms of product yield and reaction time.

**Scheme 2:**
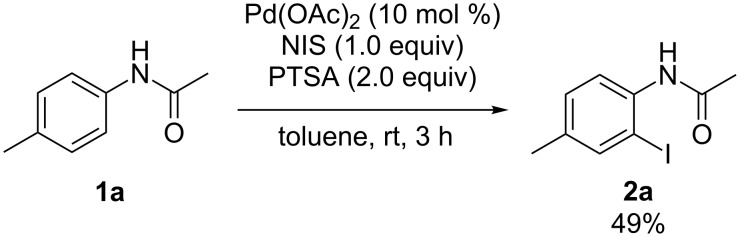
Palladium-catalyzed *ortho*-iodination of **1a** in toluene.

To illustrate the superiority of the ball-milling technique, the reaction was also investigated in an organic solvent. The reaction of **1a** with NIS conducted in toluene at room temperature for 3 h provided the desired product **2a** in only 49% yield, which was inferior to those obtained by our mechanochemical approaches (Scheme 2).

**Scheme 3:**
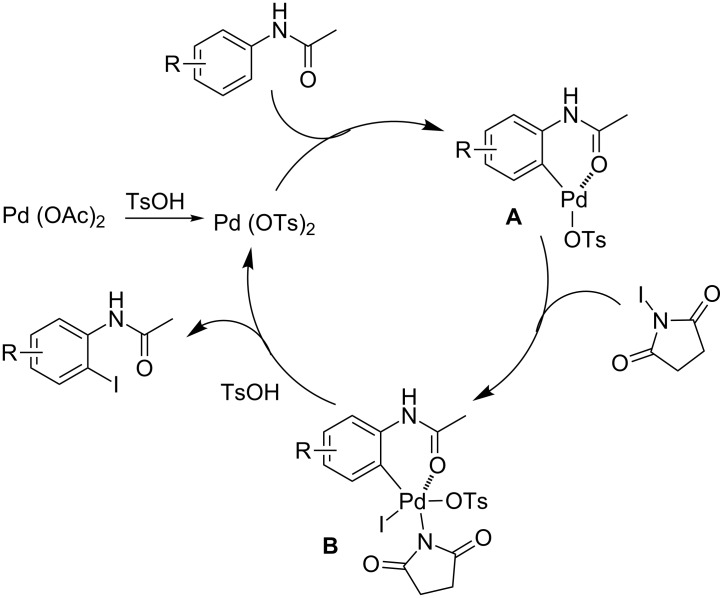
Plausible mechanism.

The plausible mechanism is proposed and depicted in Scheme 3. The addition of PTSA was essential for the present reaction. It is believed the more active Pd(OTs)_2_ is formed in situ from Pd(OAc)_2_ and TsOH [46–47]. The formed Pd(OTs)_2_ inserts into the *ortho* C–H bond of the anilides after coordination to the oxygen atom of the amide moiety, affording the species **A**. Oxidative addition of the species **A** with NIS generates the Pd(IV) complex **B**. Finally, the iodinated product is provided by reductive elimination along with regeneration of Pd(OTs)_2_ in the presence of TsOH.

**Scheme 4:**
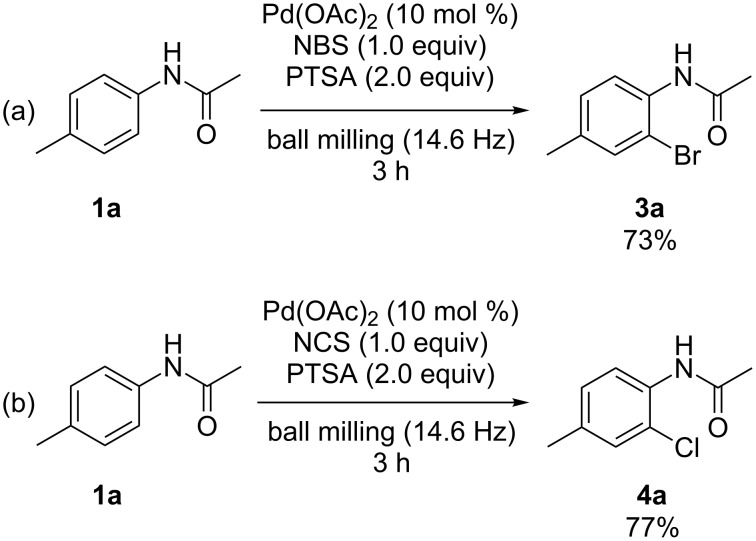
Palladium-catalyzed *ortho*-bromination and chlorination of **1a** in a ball mill.

It was intriguing to find that *N*-bromosuccinimide (NBS) and *N*-chlorosuccinimide (NCS) could also be used as reaction partners to react with the representative acetanilide **1a** under identical ball-milling conditions. The corresponding *ortho*-brominated and *ortho*-chlorinated products **3a** and **4a** were obtained in 73% and 77% yields, respectively (Scheme 4).

## Conclusion

In summary, we have developed a solvent-free and efficient protocol to synthesize *ortho*-iodinated acetanilide derivatives with Pd(OAc)_2_ as the catalyst and *N*-iodosuccinimide as the halogen source under mechanical milling conditions. This protocol shows its advantages in terms of high regioselectivity, simple operation and environmentally friendliness. In addition, the present protocol can be extended to the synthesis of *ortho*-brominated and chlorinated acetanilides delivering good yields by using the corresponding *N*-halosuccinimides.

## Supporting Information

File 1 Experimental, analytical data and NMR spectra of **2a**–**j**, **3a** and **4a**.
