# Supplementary material for: Palladium-catalyzed ortho-halogenations of acetanilides with N-halosuccinimides via direct sp2 C–H bond activation in ball mills
Source: Beilstein J Org Chem. 2018 Feb 16;14:430–5. doi: 10.3762/bjoc.14.31 (PMC5827707; doi:10.3762/bjoc.14.31)

Supporting Information

for

Palladium-catalyzed *ortho*-halogenations of acetanilides with *N*-halosuccinimides via direct sp^2 C–H bond activation in ball mills

Zi Liu[1], Hui Xu[1] and Guan-Wu Wang*[1,2]

Address: [1]Hefei National Laboratory for Physical Sciences at Microscale and Department of Chemistry, University of Science and Technology of China, Hefei, Anhui 230026, P. R. China and [2]State Key Laboratory of Applied Organic Chemistry, Lanzhou University, Lanzhou, Gansu 730000, P. R. China

Email: Guan-Wu Wang - gwang@ustc.edu.cn

*Corresponding author

Experimental, analytical data and NMR spectra of 2a–j, 3a and 4a

Table of Contents

Table of Contents	Page
Experimental	S2
Analytical data of compounds **2a–j**, **3a** and **4a**	S2–S4
References	S4
NMR spectra of compounds **2a–j**, **3a** and **4a**	S5–S14

Experimental

General methods

Nuclear magnetic resonance spectra were acquired on a BRUKER 400 AV spectrometer (400 MHz and 100 MHz for ^{1}H NMR and ^{13}C NMR, respectively). All ^{1}H NMR spectra are reported in parts per million (ppm) relative to TMS. All ^{13}C NMR spectra were reported in ppm relative to residual $CHCl_3$ (77.16 ppm) and were obtained with ^{1}H-decoupling. Data for ^{1}H NMR are described as following: chemical shift (δ in ppm), multiplicity (s, singlet; d, doublet; t, triplet; brs, broad signal), coupling constant (Hz), integration. High resolution mass spectra were obtained on a Thermo Finnigan LCQ Advantage MAX.

Materials

Solvents, toluene and acetone were purchased from Sinopharm Chemical Reagent Co., Ltd. (China) and used as received. Chemicals purchased from Sinopharm Chemical Reagent Co., Ltd. (China), Energy Chemical, J&K, Alfa Aesar, Aladdin were used without further purification. Acetanilides were synthesized according to the literature procedure [1].

General procedure for synthesis of 2. A mixture of acetanilide **1** (0.4 mmol), NIS (90.0 mg, 0.4 mmol), $Pd(OAc)_2$ (9.0 mg, 0.04 mmol), and PTSA (152.0 mg, 0.8 mmol) was added to a 3 mL stainless-steel jar with a stainless-steel ball of 5 mm diameter. The vessel was sealed and vibrated in a Spex SamplePrep 8000 Mixer Mill at a frequency of 875 cycles per minute at room temperature for 3 h. The same reaction was repeated again. Then, the reaction mixtures from two runs were washed with acetone and collected into a round–bottomed flask together with silica gel and concentrated under reduced pressure. The residue was purified by column chromatography over silica gel (ethyl acetate/petroleum ether = 1:3) to afford the corresponding product **2**.
Products **3a** and **4a** were synthesized by the general procedure for the synthesis of **2** except that NIS was replaced by NBS and NCS, respectively.

Analytical data of compounds 2a–j, 3a and 4a

N-(2-Iodo-4-methylphenyl)acetamide (2a)

^{1}H NMR (400 MHz, $CDCl_3$): δ 7.99 (d, J = 8.1 Hz, 1H), 7.60 (s, 1H), 7.35 (brs, 1H), 7.13 (d, J = 8.1 Hz, 1H), 2.27 (s, 3H), 2.21 (s, 3H); ^{13}C NMR (100 MHz, $CDCl_3$): δ 168.3, 139.1, 136.2, 135.9, 130.0, 122.3, 90.4, 24.8, 20.4. The analytical data are in accordance with the literature [2].

***N*-(2-Iodo-5-methylphenyl)acetamide (2b)**

^{1}H NMR (400 MHz, $CDCl_3$): δ 8.03 (s, 1H), 7.62 (d, J = 7.9 Hz, 1H), 7.36 (brs, 1H), 6.68 (d, J = 7.9 Hz, 1H), 2.32 (s, 3H), 2.22 (s, 3H); ^{13}C NMR (100 MHz, $CDCl_3$): δ 168.3, 139.8, 138.4, 138.1, 127.2, 123.0, 86.1, 24.9, 21.4. The analytical data are in accordance with the literature [2].

***N*-(2-Iodo-4,5-dimethylphenyl)acetamide (2c)**

^{1}H NMR (400 MHz, $CDCl_3$): δ 7.90 (s, 1H), 7.52 (s, 1H), 7.28 (brs, 1H), 2.22 (s, 3H), 2.21 (s, 3H), 2.17 (s, 3H); ^{13}C NMR (100 MHz, $CDCl_3$): δ 168.3, 139.1, 138.2, 136.1, 135.2, 123.7, 86.8, 24.8, 19.8, 19.0; HR-MS (+EI) calcd for $C_{10}H_{12}INO$ (M^+) 288.9964, found 288.9970.

***N*-(2-Iodophenyl)acetamide (2d)**

^{1}H NMR (400 MHz, $CDCl_3$): δ 8.18 (d, J = 7.8 Hz, 1H), 7.77 (d, J = 7.5 Hz, 1H), 7.43 (brs, 1H), 7.33 (td, J = 7.8, 1.3 Hz, 1H), 6.84 (t, J = 7.5 Hz, 1H), 2.23 (s, 3H); ^{13}C NMR (100 MHz, $CDCl_3$): δ 168.3, 138.9, 138.4, 129.4, 126.1, 122.3, 90.1, 24.9. The analytical data are in accordance with the literature [2].

***N*-(4-Fluoro-2-iodo-5-methylphenyl)acetamide (2e)**

^{1}H NMR (400 MHz, $CDCl_3$): δ 7.95 (d, J = 7.4 Hz, 1H), 7.40 (d, J = 8.5 Hz, 1H), 7.25 (brs, 1H), 2.24 (d, J = 1.8 Hz, 3H), 2.22 (s, 3H); ^{13}C NMR (100 MHz, $CDCl_3$): δ 168.4, 157.7 (d, J = 246 Hz), 134.4, 126.2 (d, J = 18 Hz), 124.9 (d, J = 5 Hz), 124.7 (d, J = 27 Hz), 86.0 (d, J = 8 Hz), 24.7, 14.8 (d, J = 2.8 Hz); HR-MS (+EI) calcd for C_9H_9FINO (M^+) 292.9713, found 292.9708.

***N*-(4-Chloro-2-iodo-5-methylphenyl)acetamide (2f)**

^{1}H NMR (400 MHz, $CDCl_3$): δ 8.10 (s, 1H), 7.72 (s, 1H), 7.32 (brs, 1H), 2.33 (s, 3H), 2.23 (s, 3H); ^{13}C NMR (100 MHz, $CDCl_3$): δ 168.3, 137.9, 137.6, 137.0, 130.6, 123.7, 86.1, 24.9, 20.3; HR-MS (+EI) calcd for C_9H_9ClINO (M^+) 308.9417, found 308.9418.

***N*-(5-Chloro-2-iodo-4-methylphenyl)acetamide (2g)**

^{1}H NMR (400 MHz, $CDCl_3$): δ 8.23 (s, 1H), 7.62 (s, 1H), 7.34 (brs, 1H), 2.29 (s, 3H), 2.22 (s, 3H); ^{13}C NMR (100 MHz, $CDCl_3$): δ 168.3, 139.9, 137.1, 135.4, 134.0, 122.3, 87.0, 24.9, 19.3; HR-MS (+EI) calcd for C_9H_9ClINO (M^+) 308.9417, found 308.9412.

***N*-(5-Chloro-2-iodophenyl)acetamide (2h)**

^{1}H NMR (400 MHz, $CDCl_3$): δ 8.31 (s, 1H), 7.66 (d, J = 8.5 Hz, 1H), 7.42 (brs, 1H), 6.85 (dd, J = 8.5, 2.4 Hz, 1H), 2.24 (s, 3H); ^{13}C NMR (100 MHz, $CDCl_3$): δ 168.3, 147.8, 139.3, 135.5, 126.1, 121.8, 86.6, 25.0. The analytical data are in accordance with the literature [3].

***N*-(4-Bromo-2-iodo-5-methylphenyl)acetamide (2i)**

^{1}H NMR (400 MHz, $CDCl_3$): δ 8.13 (s, 1H), 7.89 (s, 1H), 7.32 (brs, 1H), 2.36 (s, 3H), 2.23 (s, 3H); ^{13}C NMR (100 MHz, $CDCl_3$): δ 168.3, 140.8, 139.5, 137.6, 123.6, 120.3, 86.4, 25.0, 23.0; HR-MS (+EI) calcd for C_9H_9BrINO (M^+) 352.8912, found 352.8915.

N-(4-Acetyl-2-iodophenyl)acetamide (2j)

^{1}H NMR (400 MHz, $CDCl_3$): δ 8.42 (d, J = 8.6 Hz, 1H), 8.39 (d, J = 1.9 Hz, 1H), 7.92 (dd, J = 8.6, 1.9 Hz, 3H), 7.65 (brs, 1H), 2.56 (s, 3H), 2.28 (s, 3H); ^{13}C NMR (100 MHz, $CDCl_3$): δ 195.6, 168.5, 142.2, 139.2, 134.2, 130.0, 120.3, 89.1, 26.5, 25.2. The analytical data are in accordance with the literature [4].

N-(4-acetyl-2-bromophenyl)acetamide (3a)

^{1}H NMR (400 MHz, $CDCl_3$): δ 8.16 (d, J = 8.3 Hz, 1H), 7.51 (brs, 1H), 7.35 (s, 1H), 7.11 (d, J = 8.3 Hz, 1H), 2.30 (s, 3H), 2.22 (s, 3H). The analytical data are in accordance with the literature [5].

N-(2-chloro-4-methylphenyl)acetamide (4a)

^{1}H NMR (400 MHz, $CDCl_3$): δ 8.19 (d, J = 8.3 Hz, 1H), 7.53 (brs, 1H), 7.17 (s, 1H), 7.06 (d, J = 8.3 Hz, 1H), 2.29 (s, 3H), 2.22 (s, 3H). The analytical data are in accordance with the literature [5].

References

1. Gao, Y.; Huang, Y.; Wu, W.; Huang, K.; Jiang, H. *Chem. Commun.* **2014**, *50*, 8370-8373.
2. Kathiravan, S.; Nicholls, I. A. *Chem. Eur. J.* **2017**, *23*, 7031-7036.
3. Pascanu, V.; Carson, F.; Solano, M. V.; Su, J.; Z, X.; Johansson, M.; Martín-Matute, B. *Chem. Eur. J.* **2016**, *22*, 3729-3737.
4. Kotha, S.; Shah, V. R. *Eur. J. Org. Chem.* **2008**, 1054-1064.
5. Bedford, R. B.; Mitchell, C. J.; Webster, R. L. *Chem. Commun.* **2010**, *46*, 3095-3097.

^{1}H NMR (400 MHz, $CDCl_3$) of compound 2a

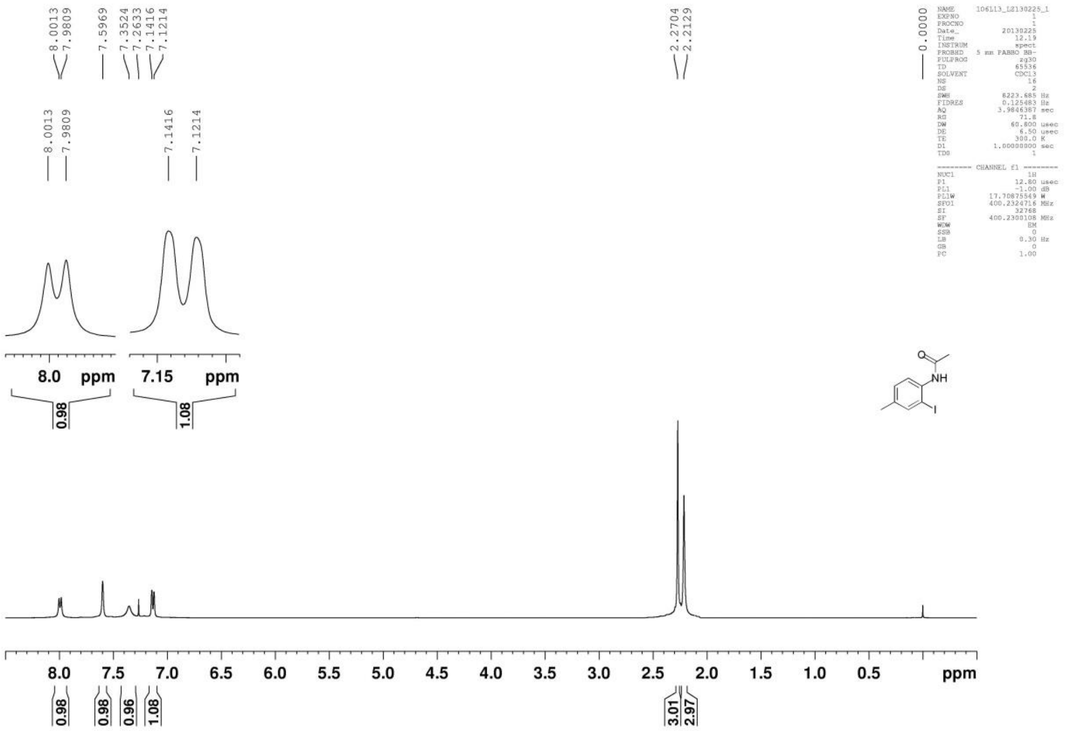

^{13}C NMR (100 MHz, $CDCl_3$) of compound 2a

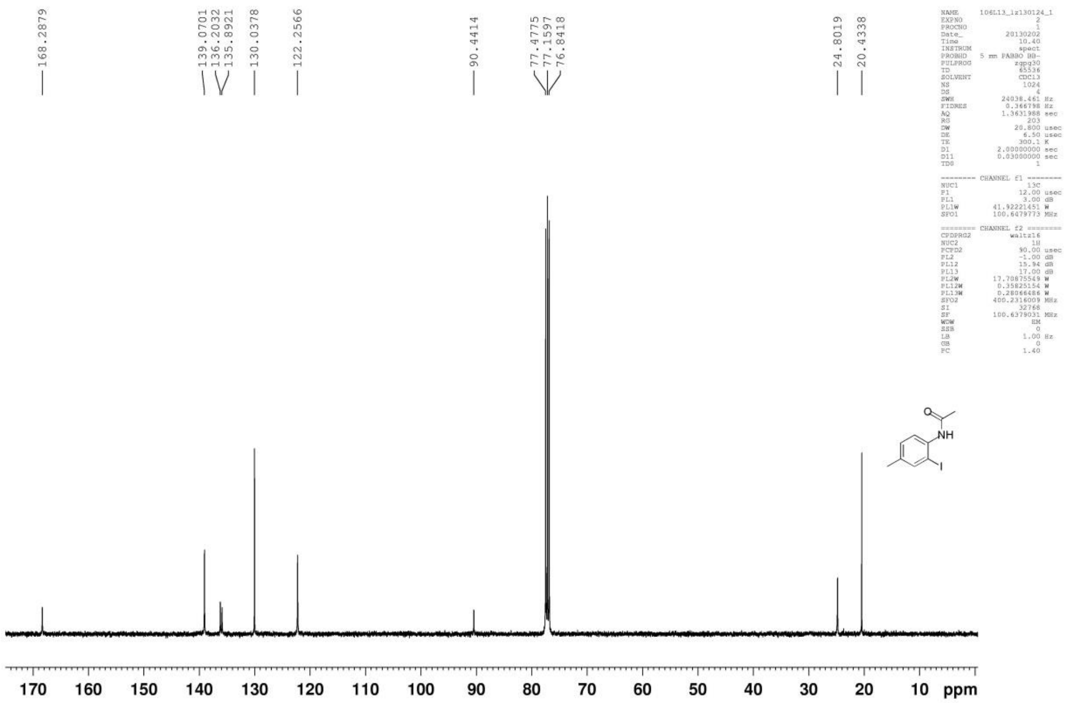

^{1}H NMR (400 MHz, $CDCl_3$) of compound 2b

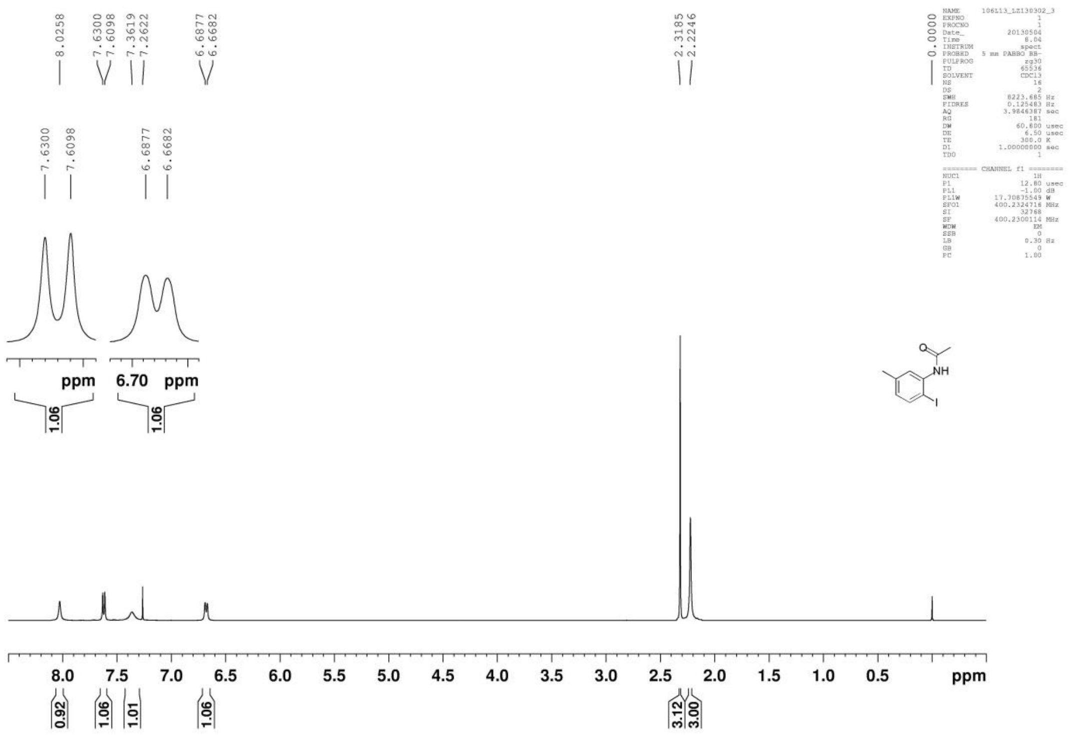

^{13}C NMR (100 MHz, $CDCl_3$) of compound 2b

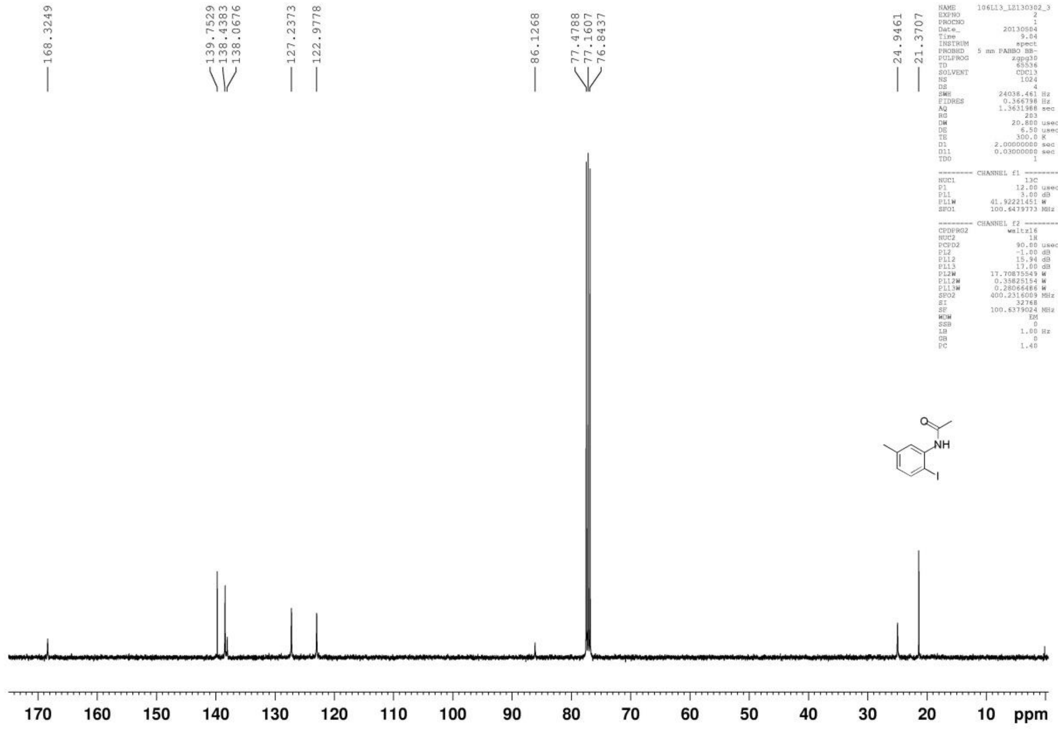

^{1}H NMR (400 MHz, $CDCl_3$) of compound 2c

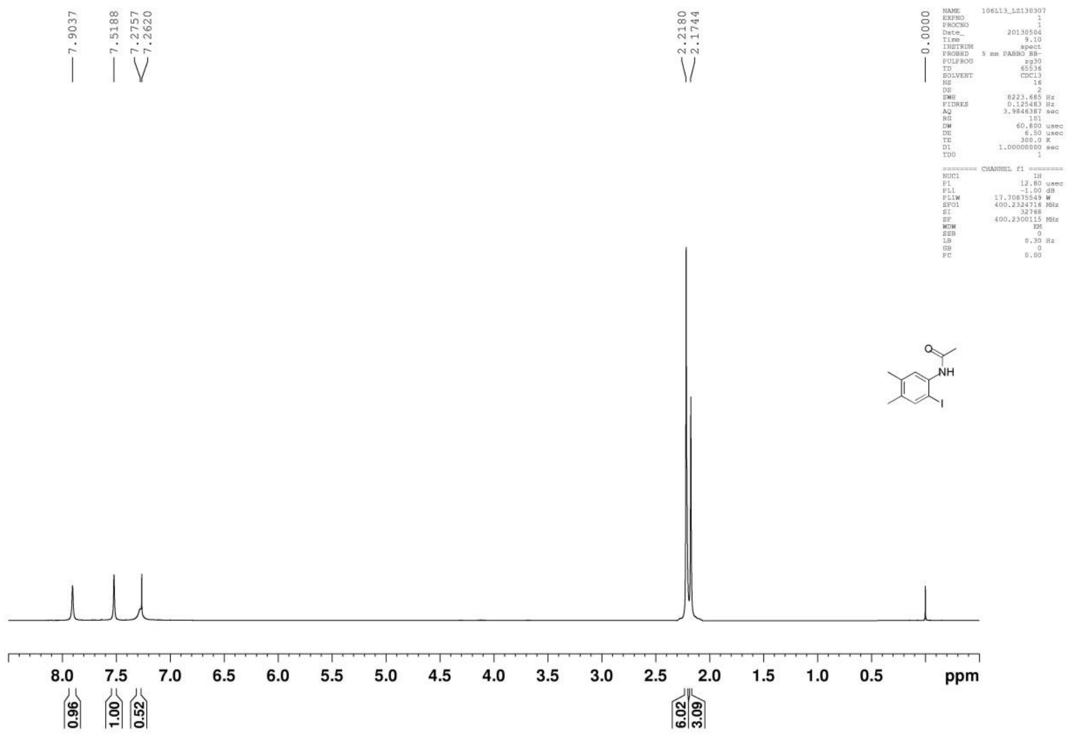

^{13}C NMR (100 MHz, $CDCl_3$) of compound 2c

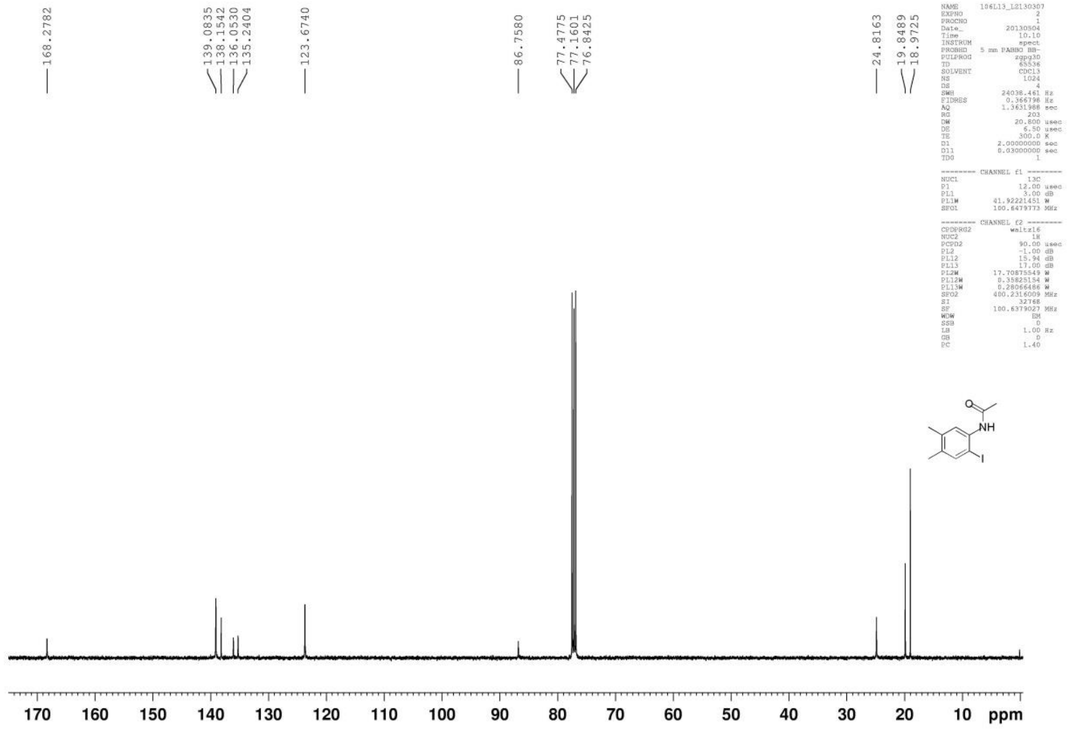

^{1}H NMR (400 MHz, $CDCl_3$) of compound 2e

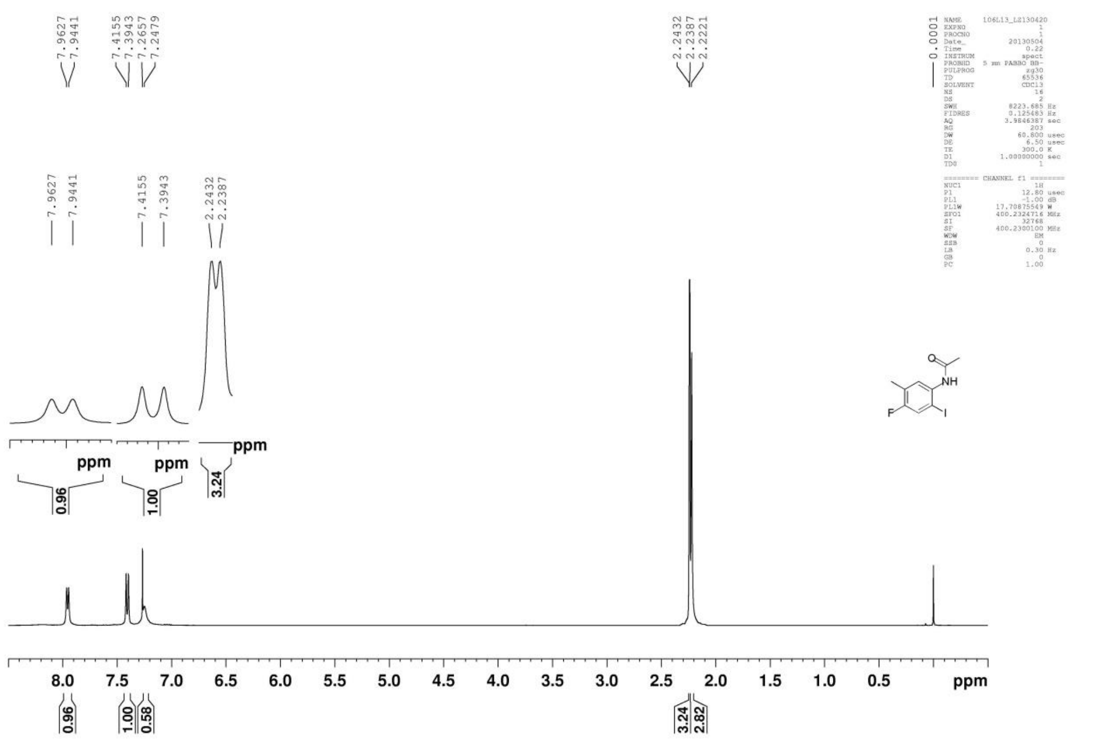

^{13}C NMR (100 MHz, $CDCl_3$) of compound 2e

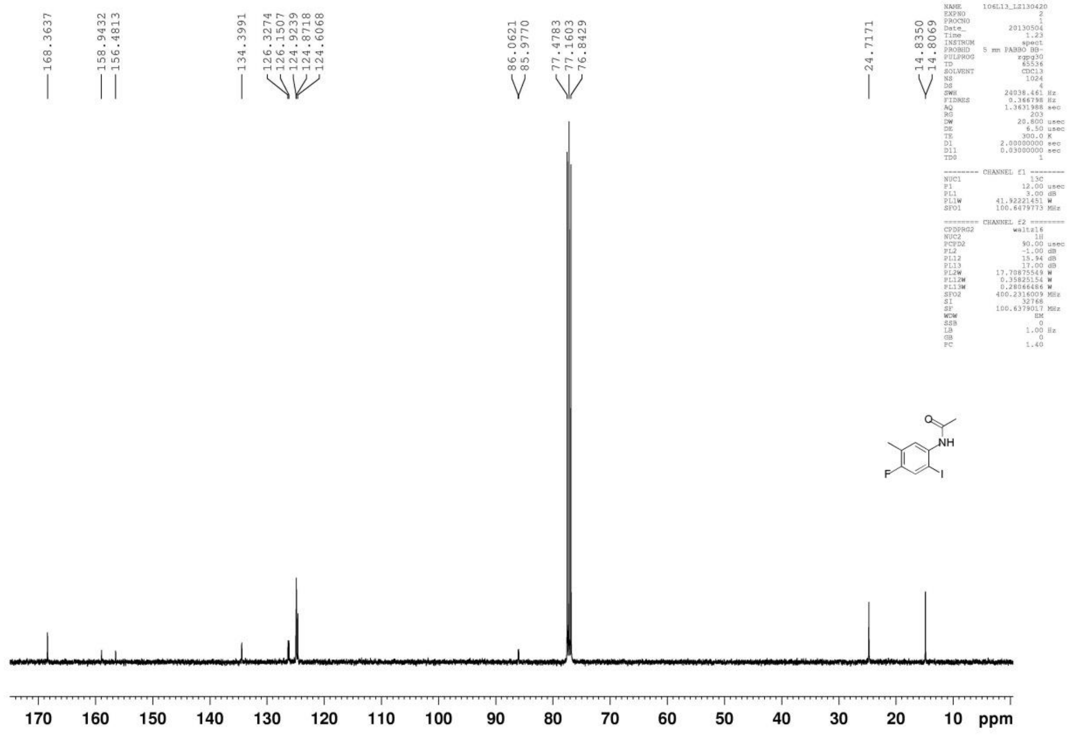

^{1}H NMR (400 MHz, $CDCl_3$) of compound 2f

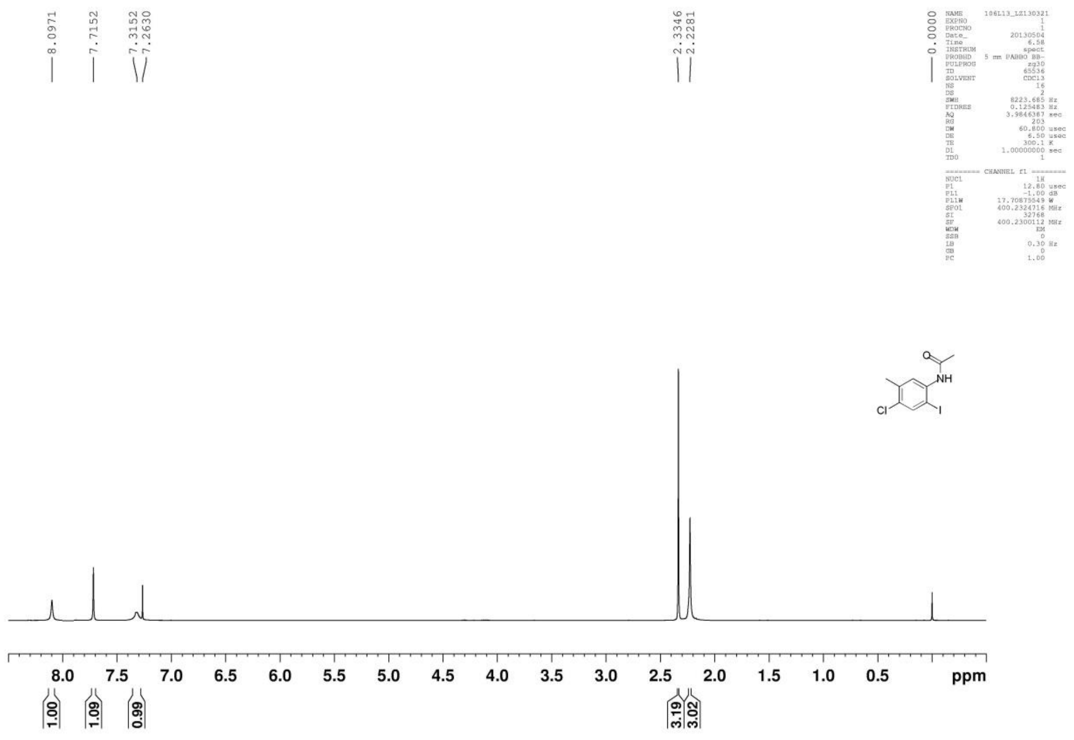

^{13}C NMR (100 MHz, $CDCl_3$) of compound 2f

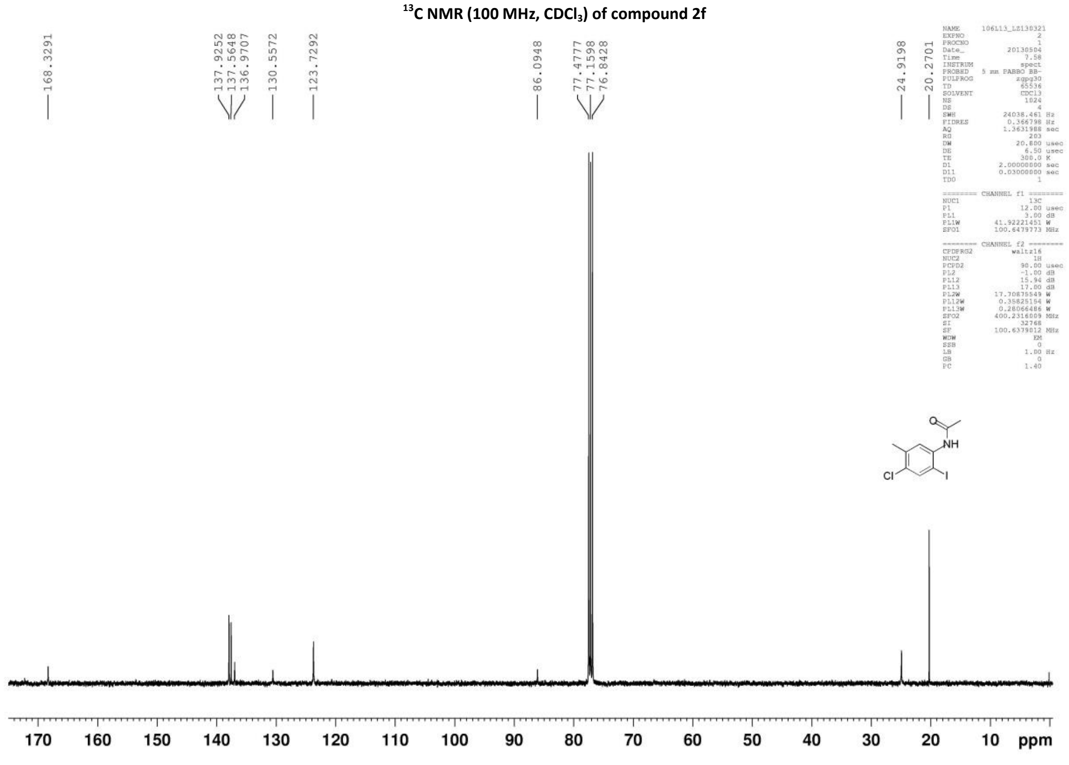

^{1}H NMR (400 MHz, $CDCl_3$) of compound 2g

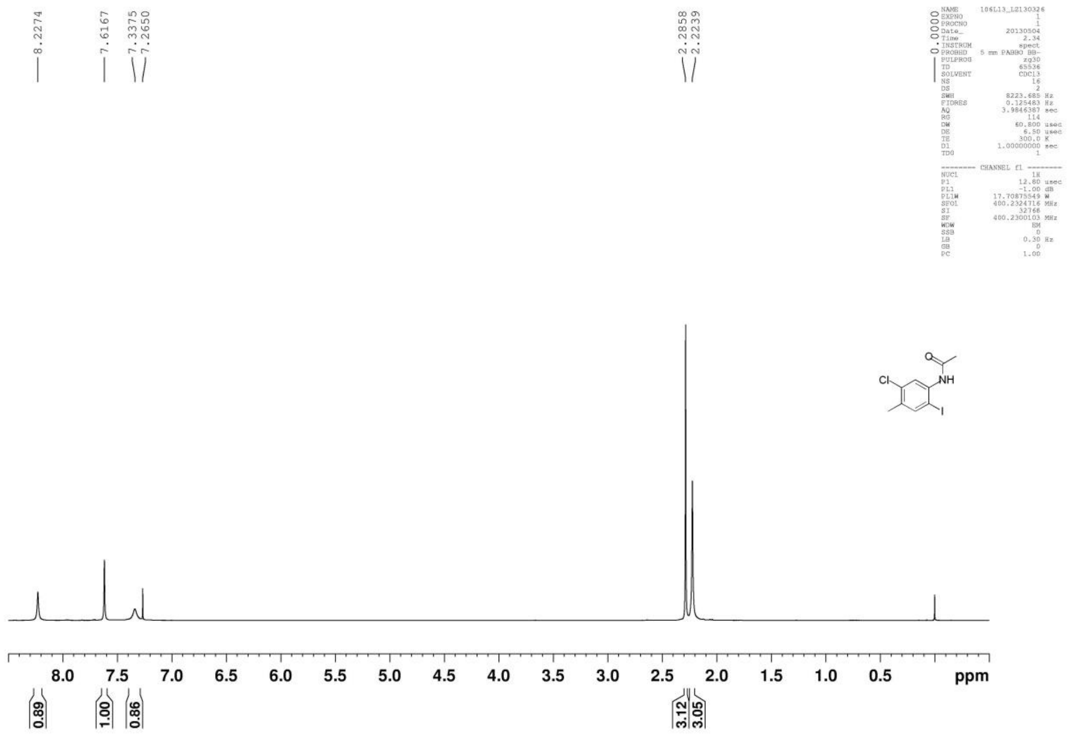

^{13}C NMR (100 MHz, $CDCl_3$) of compound 2g

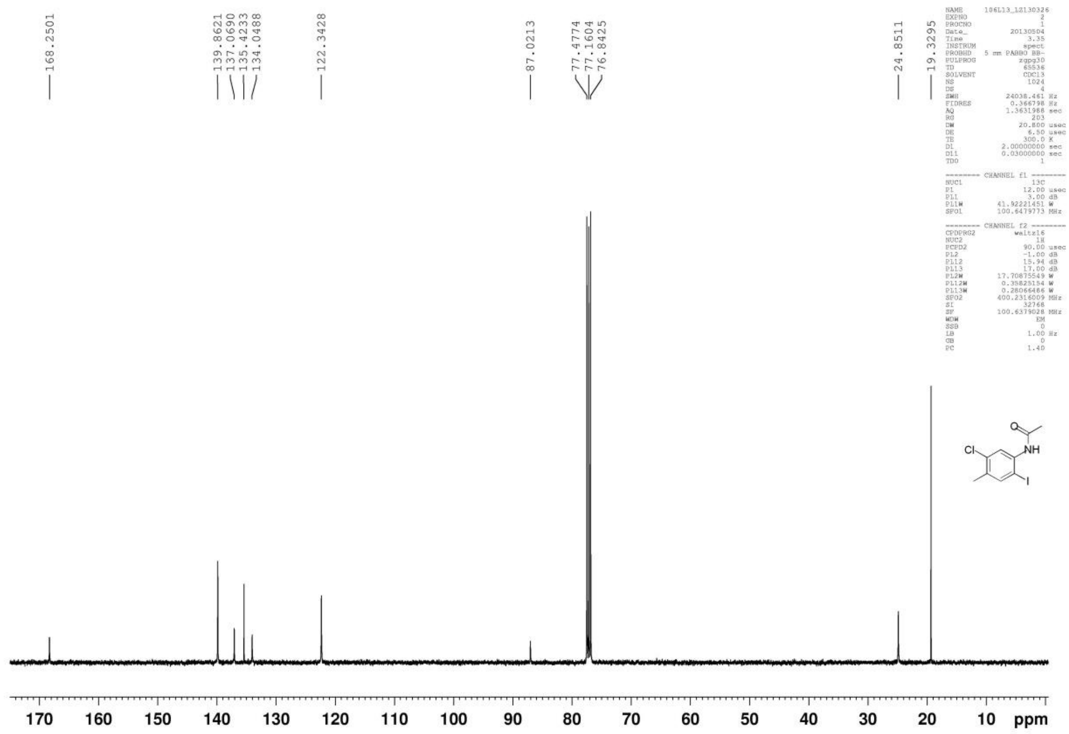

^{1}H NMR (400 MHz, $CDCl_3$) of compound 2h

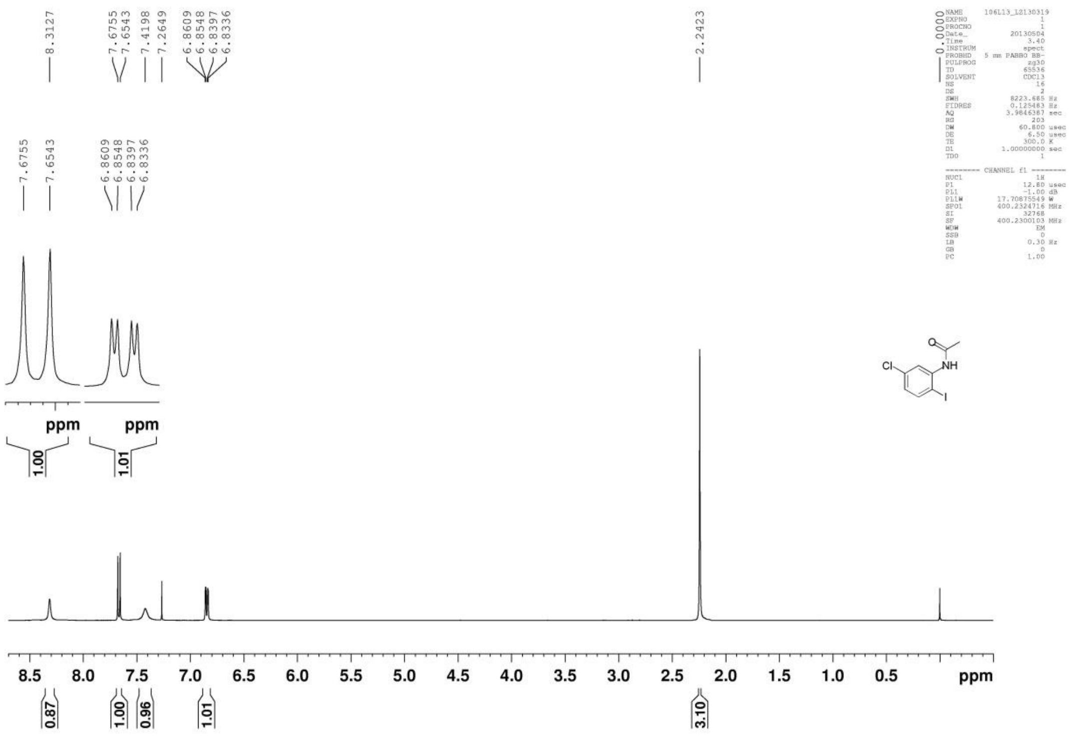

^{13}C NMR (100 MHz, $CDCl_3$) of compound 2h

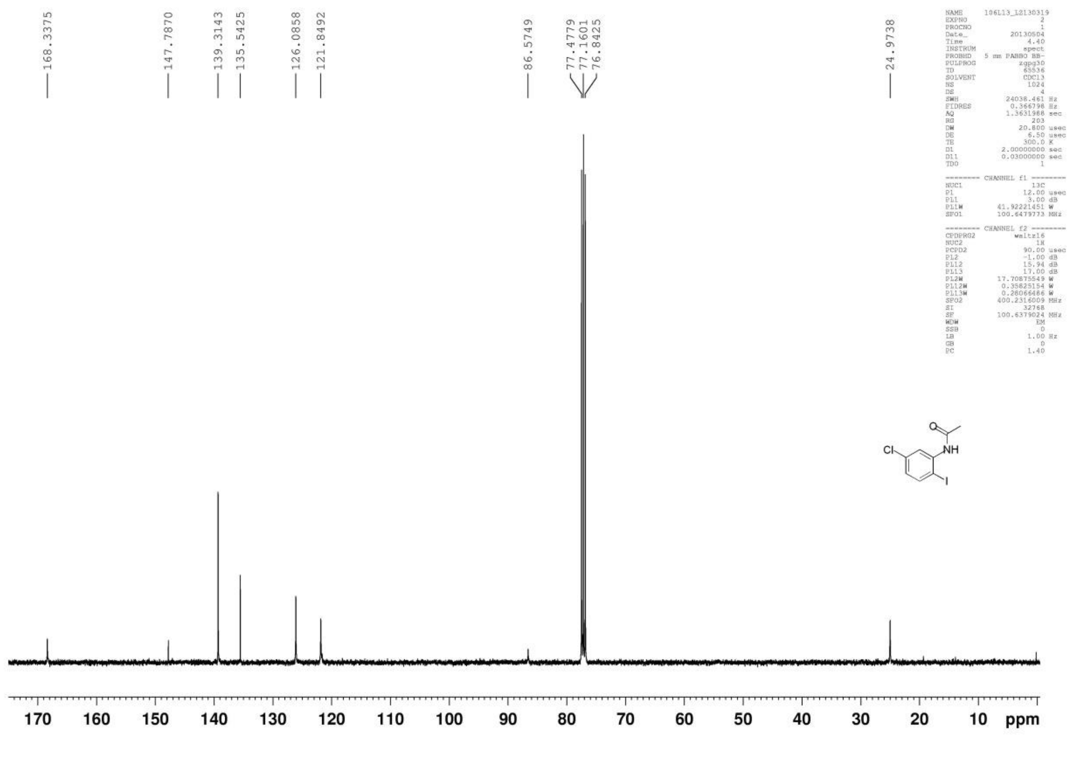

^{1}H NMR (400 MHz, $CDCl_3$) of compound 2i

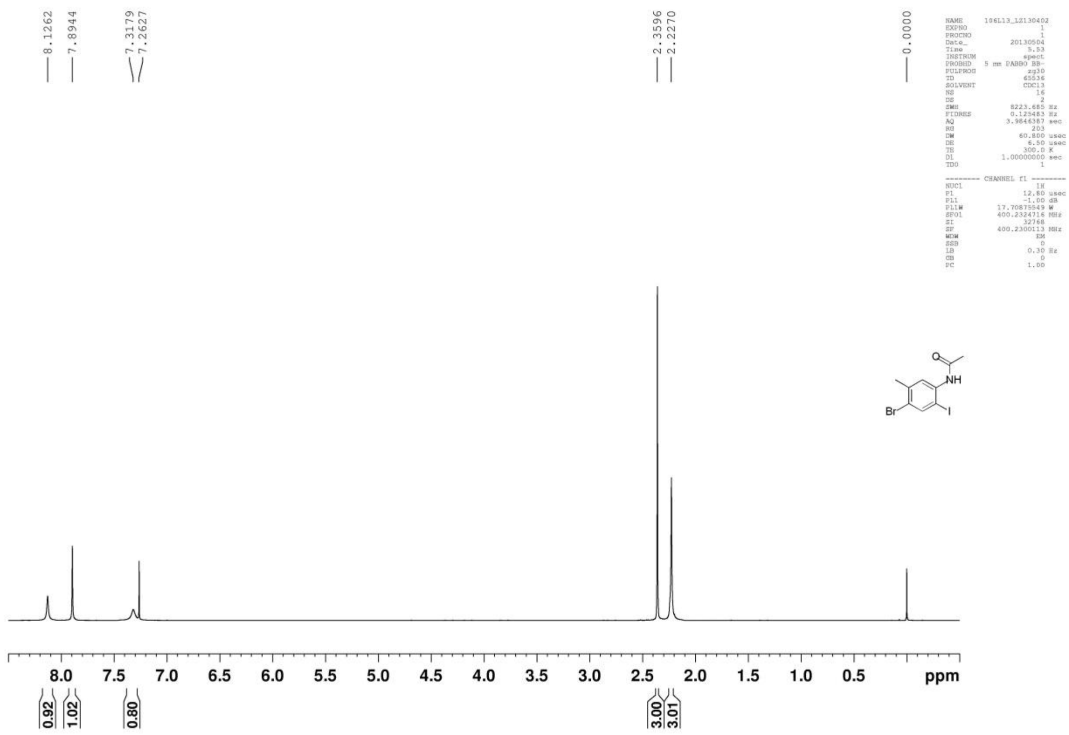

^{13}C NMR (100 MHz, $CDCl_3$) of compound 2i

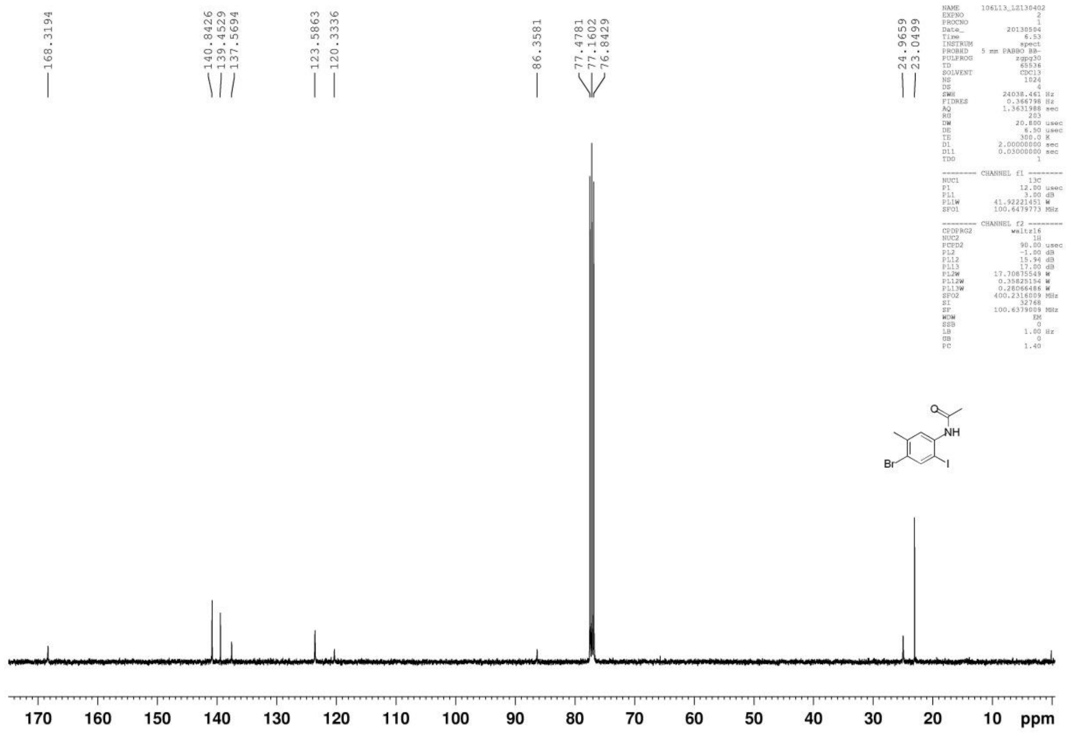

^{1}H NMR (400 MHz, $CDCl_3$) of compound 2j

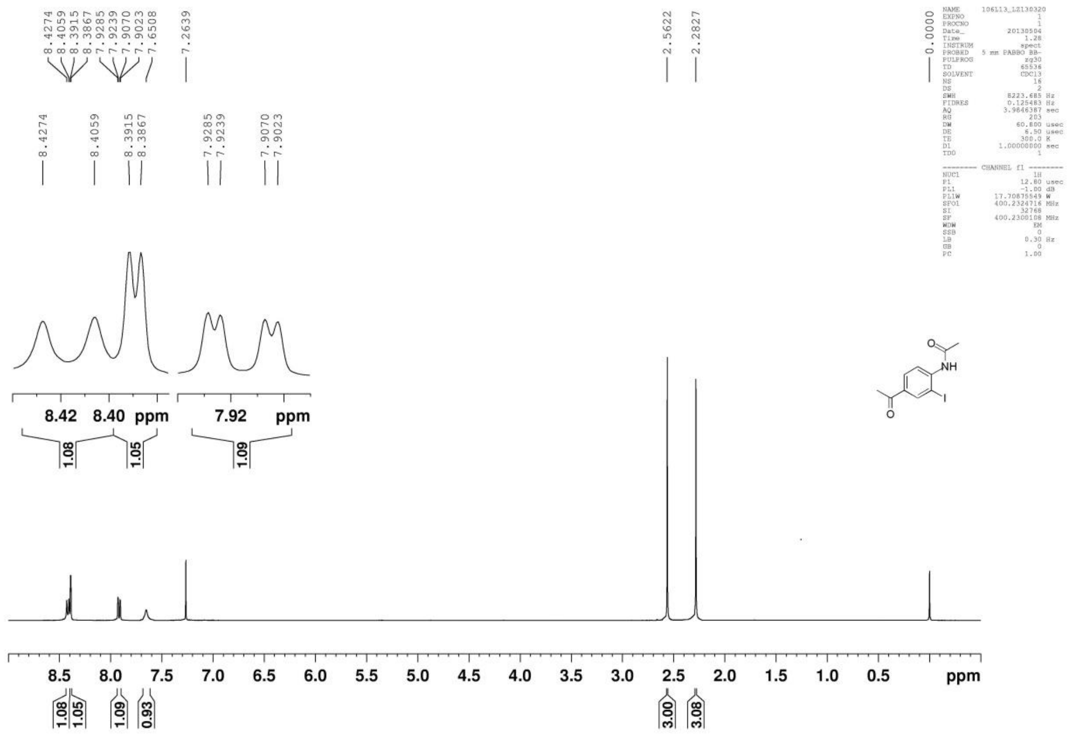

^{13}C NMR (100 MHz, $CDCl_3$) of compound 2j

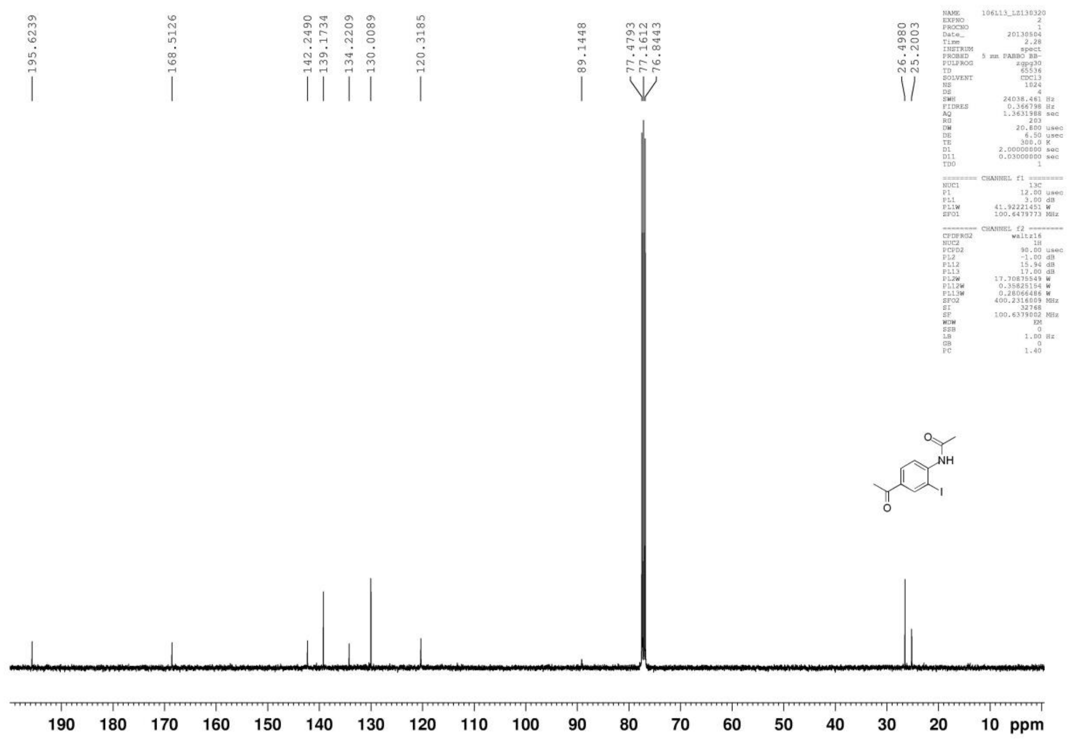

^{1}H NMR (400 MHz, $CDCl_3$) of compound 3a

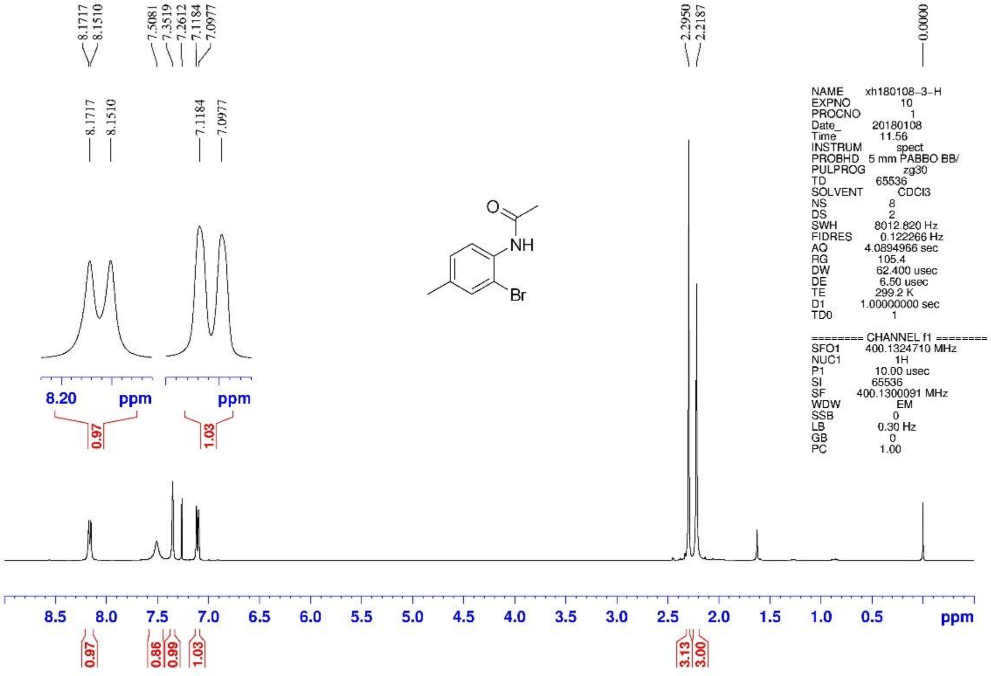

^{1}H NMR (400 MHz, $CDCl_3$) of compound 4a

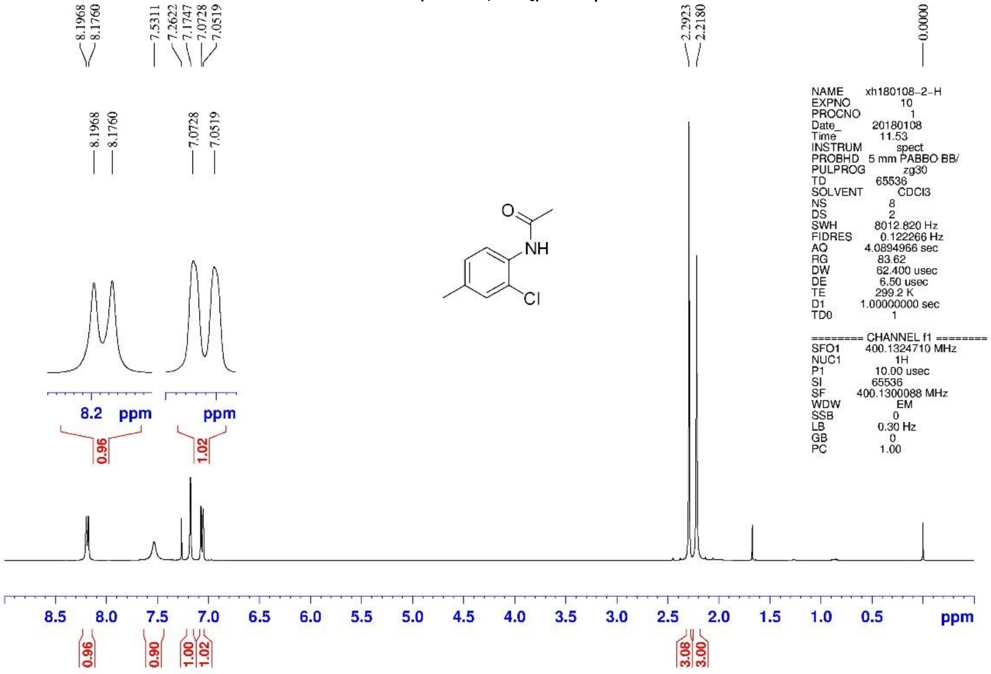

Supplement: File 1 — Experimental, analytical data and NMR spectra of 2a–j, 3a and 4a. [file Beilstein_J_Org_Chem-14-430-s001.pdf]
